# Regulation of dorso‐ventral polarity by the nerve cord during annelid regeneration: A review of experimental evidence

**DOI:** 10.1002/reg2.78

**Published:** 2017-06-13

**Authors:** Bénoni Boilly, Yolande Boilly‐Marer, Alexandra E. Bely

**Affiliations:** ^1^ UFR de BiologieUniversité de Lille59655 Villeneuve d'AscqFrance; ^2^ Department of BiologyUniversity of MarylandCollege ParkMDUSA

**Keywords:** annelid, blastema, dorso‐ventral polarity, nervous system, regeneration

## Abstract

An important goal for understanding regeneration is determining how polarity is conferred to the regenerate. Here we review findings in two groups of polychaete annelids that implicate the ventral nerve cord in assigning dorso‐ventral polarity, and specifically ventral identity, to the regenerate. In nereids, surgical manipulations indicate that parapodia develop where dorsal and ventral body wall territories contact. Without a nerve cord at the wound site, the regenerate differentiates no evident polarity (with no parapodia) and only dorsal identity, while with two nerve cords the regenerate develops a twinned dorso‐ventral axis (with four parapodia per segment instead of the normal two). In sabellids, a striking natural dorso‐ventral inversion in parapodial morphology occurs along the body axis and this inversion is morphologically correlated with the position of the nerve cord. Parapodial inversion also occurs in segments in which the nerve cord has been removed, even without any segment amputation. Together, these data strongly support a role for the nerve cord in annelid dorso‐ventral pattern regulation, with the nerve cord conferring ventral identity.

## 1 INTRODUCTION

The establishment of body axes is a key process in animal development. Body polarity cues provide developing tissue with positional information required for the development of normal phenotypes and the establishment of polarity thus typically occurs very early during development. Along with the anterior−posterior (AP) axis, the dorsal−ventral (DV) axis represents a key feature of the body plan of bilaterian animals. The developmental processes that establish DV polarity have been investigated intensively in the embryos of a range of bilaterians (e.g., Amiel, Henry, & Seaver, 2013; Henry & Martindale, 1994; Lambert, Johnson, Hudson, & Chan, 2016; Lowe et al., 2006; Lynch & Roth, 2011; Nakamoto, Nagy, & Shimizu, 2011; Tuazon & Mullins, 2015). Through these efforts, broadly conserved mechanisms for generating DV polarity have been identified, such as the involvement of the bone morphogenetic protein (BMP) pathway (De Robertis & Sasai, 1996; Ferguson, 1996; Lynch & Roth, 2011; Mizutani & Bier, 2008; Tuazon & Mullins, 2015) and, in spiralian bilaterians, cell−cell interactions involving the D‐quadrant (Goulding & Lambert, 2016; Lambert, 2010; Seaver, 2014).

By contrast with these intense efforts to understand DV polarity establishment during embryogenesis, relatively little attention has been focused on how DV polarity is established during regeneration. The relative paucity of such studies is probably due to the fact that, in contrast to embryogenesis (which occurs in essentially all animals), regeneration is highly variable across the animal tree (Bely & Nyberg, 2010). Furthermore, many of the most intensively studied model systems in developmental biology (e.g., *Drosophila*, *Caenorhabditis*, *Mus*) have relatively poor regenerative abilities, being incapable of regenerating most body regions. It is only relatively recently that bilaterian animals such as planarians and acoels, invertebrates long known for their extraordinary regenerative power, have begun to be intensively investigated with respect to the developmental mechanisms underlying regeneration (Brockes & Kumar, 2008; Gavino & Reddien, 2011; Molina, Salo, & Cebria, 2007; Molina et al., 2011; Ori & Watanabe, 2007; Reddien, Bermange, Kicza, & Sanchez Alvarado, 2007; Srivastava, Mazza‐Curil, van Wolfswinkel, & Reddien, 2014). Interestingly, recent molecular studies in both planarians and acoels have implicated the BMP pathway in the establishment of DV polarity during regeneration (Gavino & Reddien, 2011; Srivastava et al., 2014), supporting the idea that at least some aspects of axis polarity development during regeneration redeploy processes occurring during embryogenesis. Despite such similarities, however, the dramatically different contexts of embryogenesis (in which body axes are established de novo) and regeneration (in which new tissue must develop with body axes defined by the remaining old tissue) must require some different mechanisms for establishing polarity in the new tissue.

Just as experimental studies involving cell and tissue ablations and transplants have provided a wealth of information about the developmental mechanisms of embryogenesis, experimental studies of regeneration using surgeries, tissue removals, and grafting can provide valuable insights into the developmental processes operating during regeneration. For example, surgical manipulations in both amphibians and planarians have implicated tissue interactions, and specifically contact between dorsal and ventral body wall tissues, as being critical in blastema initiation during regeneration (Campbell & Crews, 2008; Carlson, 1974, 1975; Kato, Orii, Watanabe, & Agata, 1999, 2001). Such experimental studies of regeneration can be challenging to execute but can provide information about tissue‐level processes, even in the absence of molecular knowledge of the processes. Indeed, knowing where DV polarity molecular markers are *expressed*, for example, is distinct from understanding the *regulation* of DV patterning during regeneration. Organismal‐level experimental developmental studies are expected to elucidate the regulation of polarity, providing insights that can complement, and help provide context for, molecular studies of regeneration.

Annelids, the segmented worms, have been the subject of numerous regeneration studies over more than two centuries, and currently annelid regeneration is experiencing a resurgence of interest, with an increasing number of studies being published on this topic (reviewed in Bely, 2006, 2014; Bely, Zattara, & Sikes, 2014; Martinez‐Acosta & Zoran, 2015; Özpolat & Bely, 2016). The annelid body is primarily composed of a series of body segments, which in many species bear parapodia, body wall outgrowths often used for locomotion. At the anterior end of annelids is the prostomium, an asegmental cap of tissue at the tip of the head, and the peristomium, a segment‐like body region that bears the mouth located immediately posterior to the prostomium. At the posterior end of most annelids is the pygidium, an asegmental cap of tissue at the tip of the tail that bears the anus. Following transverse amputation, many annelid species can regenerate anterior and/or posterior segments and both of these abilities are reconstructed to be ancestral for the phylum (Bely et al., 2014; Zattara & Bely, 2016).

A series of early experimental developmental studies in two groups of marine annelids, nereid and sabellid polychaetes, have contributed important insights into how blastema polarity is assigned. This work used surgical manipulations, grafting, histology, and experimental regeneration studies to investigate the factors that regulate DV patterning during regeneration. Here, we review findings from this body of work that collectively provide strong evidence that the ventral nerve cord plays an important role in assigning DV polarity to the annelid blastema. Most of the studies we review were published several decades ago and mostly in French; it is our hope that by reviewing them here they can not only become better known but also provide an important framework for designing and interpreting future studies on this general topic, including studies aimed at elucidating the molecular basis of blastema polarity. Ultimately, we hope that this review can help to fuel further research on DV polarity establishment during regeneration, both in annelids and in other animals, leading to a better understanding of the fundamental mechanisms that confer polarity to regenerated tissues.

## 2 INSIGHTS INTO DV ESTABLISHMENT FROM NEREID POLYCHAETES

### 2.1 Morphology and regeneration capabilities of nereids

**Figure 1:**
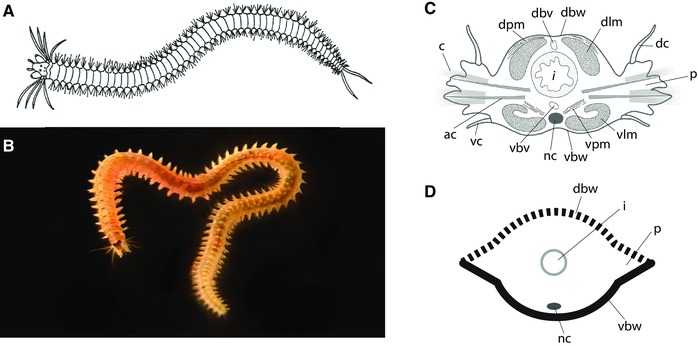
Morphology of nereid polychaetes. (A) Diagram of nereid morphology, in dorsal view with anterior to the left. The body is composed primarily of a long series of largely similar segments bearing chaetae and one pair of parapodia per segment. Reproduced with permission from Boilly et al. (1975). (B) *Nereis latescens* illustrating a typical nereid body form (this individual has small anal cirri, probably because it is regenerating a lost posterior end). Photo by Peter J. Bryant; specimen identification by Leslie H. Harris. (C) Transverse section of a segment, with dorsal side at the top and ventral side at the bottom. ac, acicula; c, chaetae; dc, dorsal cirri; dbv, dorsal blood vessel; dbw, dorsal body wall; dlm, dorsal longitudinal muscle; dpm, dorsal parapodial muscle; i, intestine; nc, nerve cord; p, parapodium; vbv, ventral blood vessel; vc, ventral cirri; vbw, ventral body wall; vlm, ventral longitudinal muscle; vpm, ventral parapodial muscle. Adapted from Boilly et al. (1975). (D) Schematic representation of a transverse section of a segment showing the dorsal body wall (dbw, dashed line), the ventral body wall (vbw, black line), nerve cord (nc), intestine (i), and parapodia (p). Parapodia are represented by triangular lateral projections with a dorsal side (dashed) and a ventral side (black). The diagrammatic representations illustrated in (D) are used in subsequent figures of this paper. Adapted from Boilly et al. (1975)

The Nereididae (ragworms) are a family of typically highly active marine polychaetes in the Errantia clade of annelids (Struck et al., 2011). The species used in the studies described here are two relatively closely related species, *Nereis pelagica* and *Hediste diversicolor* (formerly *Nereis diversicolor*; Glasby, 2015), that can be collected among *Laminaria* roots and from mud in shallow water, respectively. These two species are morphologically very similar, are typically 8−10 cm in maximum length, and have a body organization fairly typical for this family of polychaetes (Fig. 1).

The body of nereids is composed primarily of a long series of largely similar segments that bear chaetae (bristles) and well‐developed lateral parapodia used in locomotion (Fig. 1). At the anterior end, the prostomium has pigmented eyes, a pair of antennae, and palps, and the peristomium bears four pairs of tentacular cirri (some of which develop from parapodial‐like embryonic structures; Herpin, 1925). At the posterior end the pygidium bears a pair of ventral anal cirri. Within the segmented body region, external signs of DV polarity are evident in both the body wall and the parapodia. The dorsal body wall is heavily pigmented while the ventral body wall is light colored and largely devoid of pigmentation; the ventral body wall also possesses a groove that runs the entire length of the worm. The dorsal and ventral parts of the parapodia differ in several substructures (Fauvel, 1927) as well as in the length of cirri, the dorsal cirrus being much longer than the ventral cirrus (Fig. 1C). Internally, the ventral part of the body contains the nerve cord that runs just above the ventral tegument (Fig. 1C, D), a left−right pair of nephridia in each segment, and a left−right pair of longitudinal muscle bundles, the morphology of which, in transverse section, differs clearly from that of the dorsal pair (Boilly‐Marer, 1970). The nerve cord has a rope‐ladder configuration, with, in each segment, a left and a right ganglion linked via a transverse commissure and connected to adjacent segments by longitudinal connectives. The brain, which is located dorsally in the prostomium, is connected to the anterior part of the nerve cord by paired circumpharyngeal connectives. The peripheral nervous system comprises an epidermal plexus, longitudinal nerves branching off from the brain, and segmental nerves branching off from the longitudinal nerve cord connectives (Müller, 2006; Orrhage & Müller, 2005; Smith, 1957).

**Figure 2:**
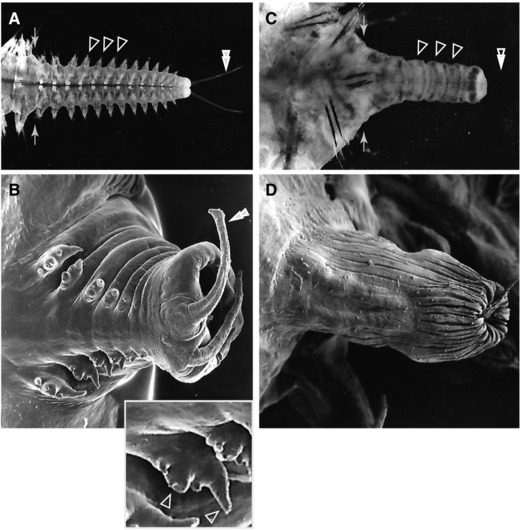
Normal and aneurogenic caudal regeneration in the nereid *Nereis pelagica*. Top panels are low magnification micrographs using epi‐illumination; bottom panels are high magnification scanning electron micrographs. Anterior is to the left. Pairs of arrows in (A) and (C) indicate the plane of amputation. (A) Normal caudal regenerate. Note the 12 new segments with parapodia (three marked by filled arrowheads) and the terminal pygidium with anal cirri (double filled arrowheads). (B) High magnification view of normal caudal regenerate with differentiated segments, in ventral view. Note the two anal cirri (one marked by filled double arrowhead) extending posteriorly from the ventral side and the longer dorsal cirri of the parapodia, both manifestations of DV polarity. The inset is a magnified view of one parapodium in the main panel; left arrowhead marks ventral cirrus and right arrowhead marks dorsal cirrus. (C) Aneurogenic caudal regenerate. The aneurogenic regenerate does not differentiate parapodia (open arrowheads) or anal cirri (open double arrowheads) but is well segmented. (D) High magnification view of aneurogenic caudal regenerate. This regenerate was induced on the dorsal side of the worm following dorsal deviation of the intestine. Note the absence of parapodia and anal cirri. In this specimen, the segmental constrictions of the body surface of the regenerate are not evident (relaxation and preservation may have obscured these), but segmentation was evident from segmental blood vessel patterns and other repeated elements prior to fixation. Images adapted from Boilly (1973)

Although nereids cannot regenerate a head, *Nereis* and *Hediste*, like many other nereids, can regenerate tail segments following transverse amputation (Bely, 2006, 2014; Boilly, 1969; Fig. 2A, B). Nereids can also regenerate parapodia if these are amputated (Combaz, 1974), a capability that, to our knowledge, has not been documented outside of this family of annelids. Importantly, surgical manipulations in nereids have shown that parapodia can be induced to form in the intersegmental region (i.e., at the boundary between segments) if the nerve cord is deviated laterally there (Combaz, 1974). A number of studies have built on this finding and have used induction of parapodia as a way to study the tissue interactions involved in regeneration, as reviewed below.

### 2.2 Contact between dorsal and ventral body wall territories induces parapodia

A series of grafting experiments have demonstrated that contact between dorsal and ventral body wall induces the formation of parapodia (Boilly‐Marer, 1969, 1971a,1971b), a process sometimes referred to as parapodial “regeneration.” In these experiments, parapodia were grafted onto different regions of the body or, alternatively, sections of body wall from different regions of the body were grafted to the dorsal or ventral side of the body, with grafts extending over several consecutive segments.

**Figure 3:**
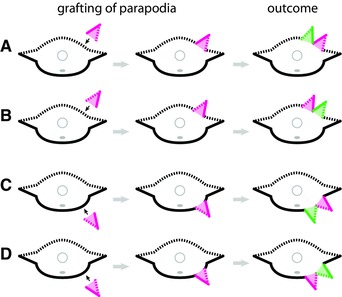
Induction of supernumerary parapodia by grafting of parapodia onto the dorsal body wall (A), (B) or ventral body wall (C), (D) in the nereid *Nereis pelagica*. Morphological features are represented as in Figure 1. Grafted parapodia are shown in pink; induced parapodia are shown in green. (A) Parapodia grafted with dorsal side down onto dorsal body wall. (B) Parapodia grafted with dorsal side up onto dorsal body wall. (C) Parapodia grafted with dorsal side down onto ventral body wall. (D) Parapodia grafted with dorsal side up onto ventral body wall. Note that, following each of the four types of grafts (A)−(D), parapodia are induced wherever dorsal and ventral tissues have been adjoined by grafting. Adapted from Boilly and Boilly‐Marer (1972)

Grafting of parapodia onto the body wall consistently induces the formation of supernumerary parapodia immediately adjacent to the grafted parapodia, regardless of the DV position of the graft (grafting onto dorsal or ventral body wall) and regardless of the DV orientation of the graft (grafting with dorsal side oriented dorsally or dorsal side oriented ventrally) (Boilly & Boilly‐Marer, 1972; Boilly‐Marer, 1969; Fig. 3). Induced parapodia always form in the same transverse plane as the site of the grafted parapodia, that is, immediately dorsal or ventral to the grafted parapodia. More specifically, parapodia are induced where dorsal and ventral tissues are juxtaposed: parapodial induction occurs where the ventral surface of a parapodium contacts dorsal tissue (Fig. 3A, B) or where the dorsal surface of a parapodium contacts ventral tissue (Fig. 3C, D). Furthermore, the induced parapodium forms with a DV orientation that is in continuity with both that of the grafted parapodium and that of the body wall (Fig. 3); no new discontinuities are established by the induced parapodium. These findings are consistent with the hypothesis that the dorsal and ventral sides of a parapodium have, respectively, dorsal and ventral identity, and that new parapodia are induced to form where dorsal and ventral tissue are juxtaposed.

**Figure 4:**
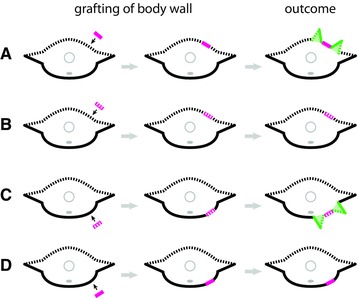
Induction of supernumerary parapodia by grafting of body wall dorsally (A), (B) or ventrally (C), (D) in the nereid *Nereis pelagica*. Morphological features are represented as in Figure 1. Body wall grafts are shown pink; induced parapodia are shown in green. (A) Ventral body wall grafted onto dorsal body wall. (B) Dorsal body wall grafted onto dorsal body wall. (C) Dorsal body wall grafted onto ventral body wall. (D) Ventral body wall grafted onto ventral body wall. Note that parapodia are induced wherever dorsal and ventral body wall tissues have been adjoined by grafting. Adapted from Boilly and Boilly‐Marer (1972)

Consistent with this hypothesis, grafting a section of body wall induces supernumerary parapodia (across the length of the graft) only when the graft and the graft site are of opposite DV polarity (Boilly & Boilly‐Marer, 1972; Boilly‐Marer, 1971a; Fig. 4). That is, parapodia are induced only when a section of dorsal body wall is grafted onto the ventral body wall or when a section of ventral body wall is grafted onto the dorsal body wall. Specifically, two supernumerary parapodia develop per segment when the graft and the graft site have opposite DV identities (Fig. 4A, C), and, conversely, no parapodia develop when the graft and the graft site are of the same polarity (Fig. 4B, D).

Together, these studies demonstrated that parapodia are induced where there is contact between dorsal and ventral body wall. Importantly, parapodia are only induced in the transverse plane corresponding to the normal parapodial plane, and this plane is where parapodial nerves are present (Boilly‐Marer, 1971b). Thus, a proposed working model for parapodial induction is that parapodia are intercalated between dorsal and ventral tegument where a nerve is present (Boilly‐Marer, 1971b). With the assumption of this model, it would be possible to determine the dorsal versus ventral nature of a piece of body wall by grafting it onto the dorsal or ventral side of the body and determining whether or not it produces parapodia, an approach taken in studies discussed below, in Section 2.4.

### 2.3 The intestine influences the nature of the regenerate

**Figure 5:**
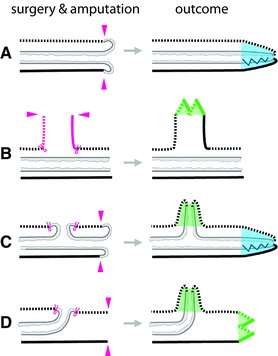
Regeneration at wound sites with and without the intestine present in the nereid *Nereis pelagica*. Surgeries and amputations are indicated in magenta in the left column. Magenta lines indicate grafted tissue, with magenta loops indicating sutures at graft boundaries. Paired magenta arrowheads indicate planes of amputation. The outcome of surgeries and amputations is shown in the right column, with blue indicating normal regenerated caudal tissue produced following simple transverse amputation (triangular points indicate parapodia on regenerated segments) and with green indicating structures induced by grafts and other surgical manipulations. (A) Normal caudal regeneration following transverse amputation. Wound healing occurs by fusion of the body wall to the intestinal wall (left) and the regenerate forms segments that develop parapodia (right). (B) Regeneration from an intestine‐less body wall graft on the dorsal body surface. Parapodia are induced at the tip of the intestine‐less fragment, where dorsal and ventral body wall come together. (Note that parapodia only rarely developed at the junction of ventral and dorsal body wall at the base of the graft [to the right of the graft, in this diagram], possibly due to the surgery disrupting innervation at this location.) (C) Dorsal tail induction following opening of the intestine on the dorsal side of the body. A segmented tail is induced dorsally but no parapodia develop in this outgrowth. Caudal regeneration proceeds normally. (D) Dorsal tail induction after deviation of the intestine to the body wall. A segmented tail is induced dorsally but no parapodia develop in this outgrowth. Caudal regeneration, which occurs in the absence of the intestine, is limited to the development of parapodia. Adapted from Boilly, Lheureux, Boilly‐Marer, and Bart (1990)

Normally, following a transverse posterior amputation, the intestine is immediately displaced outward and the severed edges of the intestinal wall rapidly adhere to the severed edges of the body wall, thereby closing the wound (Fig. 5A). A blastema is then formed, from which the pygidium differentiates first, with its two characteristic cirri growing from its ventral side. A proliferative zone then becomes evident between the regenerated pygidium and the stump. This proliferative zone generates all of the regenerated segments, which differentiate in an anterior to posterior sequence (Figs. 2A, B, 5A) (Combaz & Boilly, 1974). Even very early on in the process, morphological evidence of DV patterning is evident in the regenerate (e.g., dorsal cirri differentiate earlier than ventral cirri on developing parapodia). Segment formation during regeneration appears to be under the control of the cerebral ganglia, as when these ganglia are surgically removed the caudal regenerate is limited to only a pygidium (Boilly, 1974).

Tissue surgeries suggest that the intestine plays an important role in the formation of the caudal regenerate (Boilly, 1973). Investigating the effect of the intestine on regeneration poses significant challenges because the severed end of the intestine regenerates so rapidly. Even if amputation is accompanied by removal of the intestine over several segments, the severed intestine rapidly grows back through the segments deprived of intestine, quickly reaching the wound site and reestablishing a normal tissue configuration. The approach that was used to circumvent this problem was to completely remove the intestine from a fragment of a worm and to graft it onto the body of another worm (Fig. 5B). Without the presence of the intestine at the wound site, the wound heals by fusion of the severed ends of the body wall to each other (instead of fusion of the body wall to the intestinal wall). Under these experimental conditions, wound healing leads to the direct juxtaposition of ventral and dorsal body wall. Consistent with the working model of parapodial induction proposed above, this tissue arrangement at the wound site leads to the induction of one or two parapodia at the wound site, instead of the regeneration of segments (Fig. 5B). It is worth noting that regeneration of parapodia instead of segments has been observed in several other polychaetes (Abeloos, 1950, 1952; Boilly, 1961), although no follow‐up experiments have been made to investigate the role of the intestine in generating these regeneration phenotypes.

Although the manipulation of the intestine is difficult because of the fragility of the intestinal wall, in further experiments Boilly (1973) was able to successfully deviate the intestine to the dorsal side of the worm in order to obtain a situation in which only one side, the dorsal side, of the body wall is in contact with the intestinal wall. Following deviation of the intestine to the dorsal side of the worm in either of two different ways (Fig. 5C, D), a regenerate of caudal nature is produced at the wound site. However, although the regenerate is segmented, it develops no parapodia (Figs. 2D, 5C, D). Since the regenerate produced by these manipulations arises only from dorsal body wall, the positional quality of its body wall is assumed to be entirely dorsal; the absence of parapodia is thus again consistent with the working model for parapodial induction.

In sum, these experiments indicate that blastema initiation and differentiation is possible in the absence of the intestine as well as in the absence of ventral tissues, but the defects in the resulting blastemas suggest that both of these tissue types play important roles in normal caudal regeneration in nereids. Specifically, the defects in blastemas produced from intestine‐less wounds suggest that one important function of the intestine is in preventing fusion of body wall to body wall, and the defects in blastemas produced from all dorsal tissue implicate ventral tissues in normal blastema patterning.

### 2.4 Role of the nerve cord in blastema polarity

The experiments just described suggest that ventral tissues may be particularly important in conferring proper blastema patterning. The presence of the ventral nerve cord is one of the most significant differences between ventral and dorsal tissues, and innervation has long been implicated in regeneration in a wide range of animals (Kumar & Brockes, 2012; Pirotte, Leynen, Artois, & Smeets, 2016). For these reasons, the role of the ventral nerve cord in DV patterning and regeneration during caudal regeneration was investigated further in nereids. Specifically, studies focused on the regeneration outcome when the nerve cord was absent from the site of amputation as well as when a second nerve cord was grafted dorsally, such that two nerve cords were present at the site of amputation.

**Figure 6:**
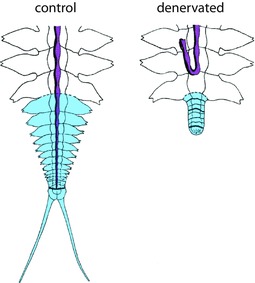
Caudal regeneration of nereids in normal animals with the nerve cord at the wound site (left) and in denervated animals in which the nerve cord is absent from the wound site (right). Regenerated tissue is shown in blue; the nerve cord is shown in purple. The regenerate of control animals includes segments with parapodia and a pygidium with anal cirri. The regenerate of denervated animals is composed of segments that do not develop parapodia and a pygidium that does not develop anal cirri. Modified from Combaz and Boilly (1974)

Boilly and Combaz (1970) found that, when the ventral nerve cord is absent from the wound site, caudal regeneration can proceed but produces a regenerate that can be considered aneurogenic (possessing no nerve cord or associated nerves) (Figs. 2C, D, 6). Although the resulting regenerate is segmented, it is shorter and narrower than a normal regenerate, has the same diameter all along its length (rather than tapering, as in normal regenerates), and displays no antero‐posterior gradient in segment differentiation (as seen in normal regenerates). These findings suggest that the nerve cord could provide positional information (e.g., anterior−posterior signals) to regenerated segments, as was suggested by Pfannenstiel (1984) based on studies in *Ophryotrocha*, a dorvilleid polychaete. In aneurogenic regenerates of nereids, no parapodia differentiate on the regenerated segments and the pygidium does not produce anal cirri (Figs. 2C, 6). In fact these aneurogenic caudal regenerates have the same general morphology as those obtained following dorsal intestine deviation (Combaz & Boilly, 1974), appearing as completely dorsalized tissue.

**Figure 7:**
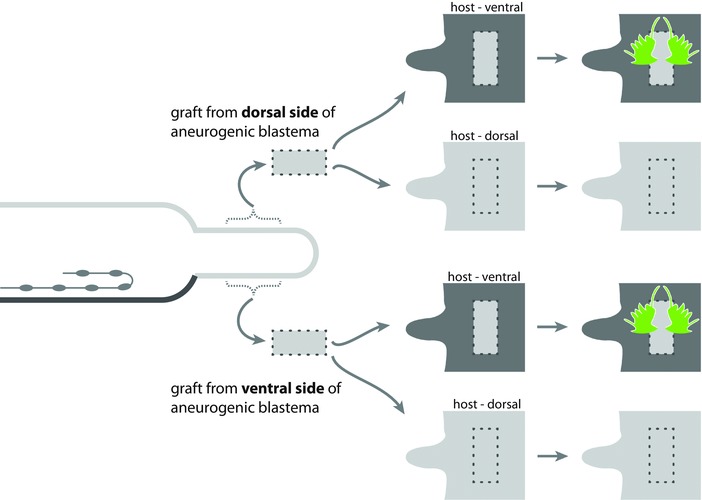
Grafting of dorsal (top two rows) or ventral (bottom two rows) body wall of aneurogenic regenerates onto the dorsal (rows 2 and 4) or ventral (rows 1 and 3) side of an intact worm. New parapodia (green) are induced on each side of the graft *only* when the graft of the body wall of the aneurogenic regenerate (whatever its localization, dorsal or ventral) is placed on the *ventral side* (dark gray) of an intact worm; no parapodia are induced when the graft is placed on the dorsal side (light gray) of an intact worm. The aneurogenic regenerate (left, generated by eliciting a blastema at a location devoid of the nerve cord, the latter having been surgically recurved) is therefore inferred to have body wall with a dorsal polarity throughout. Light gray indicates body wall with dorsal polarity; dark gray indicates body wall with ventral polarity. Dotted line indicates the perimeter of the graft. Modified from Combaz and Boilly‐Marer (1976)

The suite of regeneration abnormalities produced when regeneration is elicited in the absence of the ventral cord suggests that the resulting regenerate lacks dorso‐ventral polarity and is instead completely dorsal in nature. To evaluate the DV nature of the aneurogenic regenerate, Combaz and Boilly‐Marer (1976) grafted pieces of the ventral and dorsal body wall of these aneurogenic regenerates onto the dorsal or ventral side of normal host worms (Fig. 7). Results showed that grafting pieces of either the dorsal or ventral body wall of these aneurogenic regenerates can induce supernumerary parapodia but *only* when grafted onto the ventral side of the host worm. These findings strongly suggest that the entirety of the body wall of aneurogenic regenerates has a dorsal quality, regardless of whether it is anatomically “dorsal” or “ventral.” The dorsal quality of the ventral side of aneurogenic regenerates can also explain the observation that supernumerary parapodia occasionally develop at the junction between the ventral side of the aneurogenic regenerate and the ventral side of the stump (Combaz & Boilly, 1971). Further evidence of the dorsal quality of the body wall in aneurogenic regenerates was obtained by inducing these worms to develop into epitokes (a modified swimming form specialized for reproduction), which can be done by ablating the cerebral ganglia. In normal worms, the induction of epitoky triggers changes in the morphology of segments and especially of the pygidium, which develops anal papillae for spawning both on its dorsal side and laterally. When Boilly‐Marer and Combaz (1972) induced animals with aneurogenic regenerates to become epitokes, they found that anal papillae develop not only dorsally and laterally but also ventrally; thus the ventral side of aneurogenic regenerates responds as if it is dorsal.

Combaz (1975) attempted to further test the hypothesis that the ventral nerve cord confers a ventral quality to the regenerate by investigating caudal regeneration in the presence of two nerve cords. To test the effect of a second nerve cord, grafting the nerve cord alone into the graft area was found not to be a viable approach; such grafts did not take well and did not regenerate caudally after amputation. However, grafting the nerve cord along with its adjacent body wall onto the dorsal region of a normal host animal was able to elicit caudal regeneration, including regeneration of both the normal and the grafted nerve cord, after amputation. Transverse amputation through the body region containing two nerve cords (the normal ventral cord and the grafted dorsal one) produces a caudal regenerate with a pygidium with twice the normal number of anal cirri (four instead of two) and segments with twice the normal number of parapodia (four instead of two). The parapodia of the regenerated segments include two parapodia laterally (in the normal position) and two additional parapodia dorsally. The dorsal parapodia are separated by a zone of unpigmented body wall, resembling normal ventral body wall. Because the dorsal grafts in these experiments included not only ventral nerve cord but a small piece of associated ventral body wall as well, it is not possible to determine whether the regenerated structures that differentiate dorsally (the two supernumerary parapodia and the ventral territory) are induced by the nerve cord itself or derived from the associated ventral body wall. However, results indicate that the ventral nerve cord and/or the strip of associated ventral body wall are sufficient to induce a second DV axis in the regenerate.

**Figure 8:**
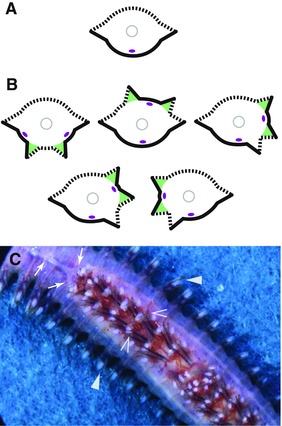
Nereids found in nature with normal and twinned DV axes, shown in transverse sections (A), (B) and in a photograph (C). Morphological features are represented as in Figure 1. (A) Section of a normal worm. (B) Sections of naturally occurring abnormal worms with twinned DV axes. Note that, in each case, individuals have two ventral territories (black lines) centered on a nerve cord (purple oval), two dorsal territories (dashed lines), and four parapodia (triangular points), each of which occurs between ventral and dorsal territories. The two parapodia that are inferred to be the induced supernumerary parapodia are shaded in green. Diagrams are based on *Nereis pelagica* L. with a doubled nerve cord in the posterior‐most 19 segments ((B), upper left); *N. pelagica* L. with a doubled nerve cord in the posterior‐most six segments ((B), upper middle); *N. pelagica* L. with a doubled nerve cord in the posterior‐most five segments ((B), upper right); *Perinereis cultrifera* G. with a doubled nerve cord in the posterior‐most 18 segments ((B), lower left); *N. pelagica* L. with a doubled nerve cord in the posterior‐most nine segments ((B), lower right). (A) and (B) are adapted from Boilly et al. (1975). (C) Image of the ventral side of the individual diagrammed in (B), upper left diagram. Anterior is to the upper left. Note the DV axis duplication beginning in the upper left corner of the picture, exactly where the nerve cord (white linear structure) bifurcates into two (arrows). In the region with twinned axes, the body has four parapodia per segment: two normal lateral ones (filled arrowheads) and two additional ones in the middle of the ventral side of the worm (open arrowheads). The small amount of body wall between the two additional parapodia has a dorsal quality, as indicated by the presence of dark pigmentation, and the length of the parapodial cirri closest to this region suggests these additional parapodia have a dorsal surface in continuity with this small strip of dorsal body wall. Photo by B. Boilly

**Figure 9:**
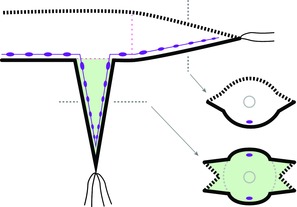
Specimen of a nereid found in nature with two regenerated tails, one of which has a twinned DV axis. Diagram of a sagittal section of the posterior end of the individual (anterior is left) is shown at the top left; diagrams of transverse sections of both tails are shown at the bottom right. This specimen of *Perinereis cultrifera* appears to have regenerated a normal tail in the original AP orientation as well as a second tail, with a twinned DV axis, ventrally (green). Magenta dotted lines indicate the inferred planes of amputation and dashed gray lines represent the position of the corresponding transverse sections. The ventral tail with a twinned DV axis has four parapodia per segment, two dorsal territories (dashed body wall), two ventral territories (black body wall), and two nerve cords (purple). See text for details. In the sagittal section, the ventral nerve cords (purple) are represented with segmental ganglia (ovals) and interganglionic connectives (lines). Note that the intestine is not shown in the sagittal section but is shown in the transverse sections. Adapted from Boilly et al. (1975)

Remarkably, worms with tails that have a double nerve cord have occasionally been found in nature (Boilly, Boilly‐Marer, & Combaz, 1975; Boilly‐Marer, 1970), possibly resulting from damage and regeneration, and in all cases the tails of such worms have the same general morphological characteristics as the double nerve cord animals produced by grafting (Fig. 8). The tails of these wild specimens have four parapodia per segment (instead of the normal two), two body wall regions with characteristic ventral attributes, and two body wall regions with characteristic dorsal attributes, with the dorsal and ventral body wall regions in continuity with the corresponding face of the parapodia. The affected region also has twice the normal number of a range of morphological features, including two pairs of ventral longitudinal muscles, two pairs of dorsal longitudinal muscles, two ventral longitudinal blood vessels, and two dorsal longitudinal blood vessels; the intestine, which runs centrally through the animal, is not doubled. In the affected region, these double nerve cord worms appear to be like a pair of worms joined along a line passing through the middle of the two dorsal territories. Double nerve cord tails have a single pygidium but two pairs of anal cirri (Fig. 9), indicating that the pygidium is morphogenetically doubled as well.

Interestingly, in animals found with naturally twinned DV axes, when the doubling of the nerve cord occurs in the middle of the ventral territory in such a way that the two nerve cords run ventro‐laterally, a dorsal territory is evident in the middle of the ventral territory (Fig. 8B, C). This observation suggests that a dorsal territory is able to differentiate from the ventral territory, and that the nature of the body wall (dorsal vs. ventral) can be reprogrammed during regeneration. This conclusion is reinforced by the observation of regeneration of a second tail ventrally after deviation of the intestine ventrally, a surgical manipulation which unavoidably severs the nerve cord (B. Boilly, unpublished data). This manipulation has a very low success rate; the surgery typically fails because it requires both a successful deviation of the intestine ventrally (a location where it is difficult to maintain the grafted tissues) and a successful regeneration of both severed ends of the nerve cord into the second tail (yet commonly the ends of the nerve cord reconnect to one another and fail to enter the second tail). However, an individual in which such a situation is thought to have occurred was fortuitously recovered from nature (Fig. 9). This specimen had two regenerated tails, one regenerated normally caudally and the second one regenerated ventrally (Boilly, Boilly‐Marer, & Combaz, 1975). The ventral tail of this specimen was a double nerve cord regenerate similar to those obtained and observed by the surgeries described above, with four parapodia per segment, two ventral territories and two dorsal territories. As this double nerve cord tail regenerates from the ventral territory, this suggests that ventral territory can differentiate into dorsal territory.

## 3 INSIGHTS INTO DV ESTABLISHMENT FROM SABELLID POLYCHAETES

### 3.1 Sabellid morphology and natural DV inversion along the AP axis

The Sabellidae (feather‐duster worms) are a large family of filter‐feeding polychaetes in the Sedentaria clade of annelids (Struck et al., 2011). These worms have an extensive crown of tentacles for feeding and construct tubes buried in sediment or attached to rocks, using their lateral parapodia to move within the tubes. The species used in the studies described here are *Sabella pavonina*, *Sabella spallanzanii* (formerly *Spirographis spallanzanii*; Read, 2015b), *Bispira melanostigma* (formerly *Sabella melanostigma*; Read, 2015a), and *Branchiomma nigromaculata*. Adults of these species are typically 10 cm or more in length and can be collected from shallow marine waters.

**Figure 10:**
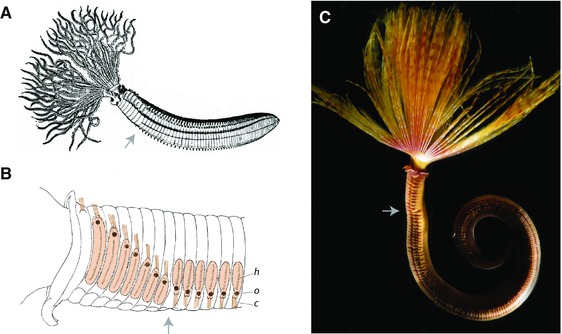
Morphology of sabellid polychaetes, shown removed from their tubes. (A) Diagram of sabellid morphology, with anterior to the left. A crown of feeding tentacles is present at the anterior end of the worm, and the main part of the segmented body is composed of a small number of thoracic segments at the anterior end followed by a larger number of abdominal segments more posteriorly. The gray arrow indicates the boundary between thoracic and abdominal segments in (A), (B), and (C). The diagram is of *Dasychone infracta*, Kr. (after Malmgren) from the *Encyclopaedia Britannica* 1911, Chaetopoda, from Wikimedia Commons. (B) Detail of the anterior end of the sabellid *Branchiomma nigromaculata* in lateral view (anterior to the left), showing the inversion in parapodial morphology between the thoracic segments (eight in total) and the abdominal segments (first six shown). Parapodial structures are highlighted in brown. In the thoracic region, the second through eighth segments have parapodia that bear chaetae pointing dorsally, an ocellus (dark spot) near the dorsal edge, and a line of hooks ventral to the ocellus (the first thoracic segment is partially fused with the collar and bears only chaetae, pointing dorsally). In the abdominal region, segments have parapodia with an inverted morphology, bearing chaetae pointing ventrally, an ocellus near the ventral edge, and a line of hooks dorsal to the ocellus. c, chaetae; o, ocellus; h, hooks. Modified from Berrill (1978) and reproduced with permission. (C) *Sabella spallanzanii*. Photo by Geoffrey B. Read

Unlike the nereids, in which segments are largely similar along the entire length of the body, segment morphology in sabellids differs markedly along the AP axis. Sabellids have two distinct segmented body regions, an anterior thoracic region (comprising four to 12 segments depending on the genus, but typically eight) and a posterior abdominal region (comprising numerous segments and ending with the pygidium) (Rouse & Pleijel, 2001) (Fig. 10). The thoracic and abdominal segments exhibit a number of morphological differences, including, remarkably, several manifested as DV inversions, as described further below. Such DV inversions are characteristic of sabellids (Knight‐Jones, 1981), and are found in the closely related serpulids as well.

One of the clearest differences between thoracic segments and abdominal segments is that parapodial morphology is dorso‐ventrally inverted between these body regions (Berrill, 1977; Knight‐Jones, 1981; Rouse & Pleijel, 2001). In thoracic segments, parapodia bear a bundle of chaetae dorsally and a row of hooks ventrally, with an eye‐shaped spot between chaetae and hooks. By contrast, in abdominal segments, although the same structures occur, the chaetal bundles occur ventrally while the hooks occur dorsally (Fig. 10B). No change in AP polarity of parapodia appears to accompany this DV polarity inversion, since all the hooks, wherever they occur, point anteriorly. This striking inversion of DV parapodial morphology has been proposed to be related to the ability of sabellids to move efficiently both forward (to expand the crown of tentacles outside the tube) and backward (to retract the tentacles and retreat from predators) in their tube (Berrill, 1977).

Another anatomical feature that displays an apparent DV inversion along the body axis in sabellids is the fecal groove (Rouse & Pleijel, 2001). The fecal groove is a deep, ciliated groove that runs along the outer body wall of the animal and is used to carry feces from the anus to the anterior extremity, necessary to avoid fouling the tube in which the animal lives. The fecal tube runs mid‐ventrally throughout the abdomen, running from the pygidium to the boundary between abdomen and thorax. At that point, however, it is deflected around the right side of the body to the dorsal side and continues there, running dorsally all the way to the anterior end of the worm. Another epidermal feature of sabellids, the ventral glandular shield (involved in tube secretion), is not deflected dorsally; in the abdomen it is divided into two tracts, with one tract running along each side of the fecal groove, and in the thorax it remains ventral, running as an undivided tract all the way to the anterior end of the worm (Berrill, 1977; Fauvel, 1927). Thus, DV polarity inversion is evident in parapodia and the fecal groove but not all external structures of sabellids.

### 3.2 Correlation between natural DV inversion and position of the ventral nerve cord

**Figure 11:**
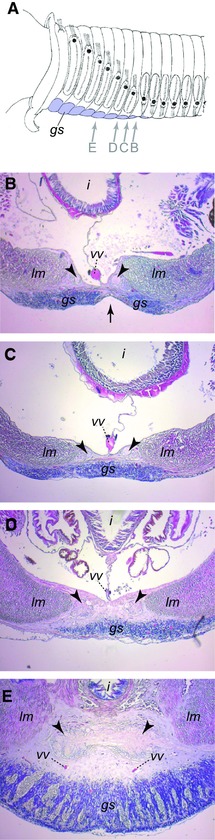
Histological sections of *Sabella spirographis* showing axial variation in the position of tissues across the transition from thorax to abdomen body regions. Views are of the ventral region of transverse sections in progressively more anterior sections, from the first abdominal segment anterior into the thoracic region. (A) Diagram indicating section locations (arrows) in the anterior region of the worm. Modified from Berrill (1978) and reproduced with permission. (B) Section in the first abdominal segment, close to the thorax/abdomen boundary. The fecal groove is present mid‐ventrally at this axial position (its normal position throughout the abdominal region) and separates the glandular shield into left and right parts. (C) Section in the last thoracic segment. The glandular shield covers the entire ventral side of the worm. (D) Section in the middle of the posterior half of the thorax. The nerve cord is separated from the glandular shield by an extracellular matrix. (E) Section in the middle of the thorax. The nerve cord is largely isolated from the glandular shield (which is much thicker here than more posteriorly) by a prominent extracellular matrix. Note that the ventral nerve cord occurs more internally and farther from the ventral body surface at position D and even more so at position E, compared to positions B and C. Tissues were fixed in 6% paraformaldehyde in filtered sea water and stained with hematoxylin/eosin. Labeling is as follows: arrowheads, individual cords of the ventral nerve cord (the ventral nerve cord comprises two separate cords joined in each segment by a nerve connection); arrow, fecal groove; i, intestine; gs, ventral glandular shield; lm, longitudinal muscles; vv, ventral vessel

Given the relationship between the nerve cord and DV polarity in nereids, described above, it is of interest to know whether the position of the nerve cord differs between the thorax and abdomen in sabellids, potentially correlating with the natural DV inversion in these animals. Histological sections of *Sabella spirographis* reveal that the position of the ventral nerve cord does indeed differ between thorax and abdomen (Fig. 11). Specifically, the nerve cord is farther from, and more isolated from, the ventral body wall in the thorax than in the abdomen. In the abdomen the nerve cord is tightly apposed to the ventral body wall, as is typical in annelids. In this body region, the ventral body wall is not covered by the glandular shield but does possess the ciliated fecal groove, running mid‐ventrally (Fig. 11B). By contrast, in the thorax, the ventral body wall is covered by the extensive and thick glandular shield but does not possess the fecal groove (this structure is located on the dorsal side in this region of the body). As a consequence, in the thorax the nerve cord is displaced somewhat dorsally, away from the ventral body wall and closer to the gut (Fig. 11C−E). In the anterior part of the thorax, the separation of the ventral body wall and the nerve cord is even greater due to the presence of a substantial extracellular matrix between the nerve cord and the glandular shield, as well as the increased thickness of the glandular shield itself in this region compared to more posterior regions (Fig. 11E). The specific configuration of the nerve cord also differs in the thorax compared to the abdomen. Specifically, in the thorax, the nerve cord is composed of two separate connective cords (hemicords), instead of one fused cord, and these separate cords run laterally along the left and right sides of the gut, close to the gut's ventral surface in the posterior part of the thorax and progressively more dorsally in more anterior regions. The anterior‐most parts of the connective cords appear to ultimately become the ventral roots of the circumpharyngeal connectives that connect to the brain (Orrhage, 1980). (Thus, the anterior part of the connective cords form a circumpharyngeal collar that ascends gradually dorsally to the brain over the entire thoracic region, as opposed to ascending only within the peristomium, as is typical in many other polychaetes.) Interestingly the position of parapodia is also increasingly dorsal from the posterior to the anterior regions of the thorax (Fig. 11A), paralleling the dorsal shift in the circumpharyngeal connectives. In summary, then, the natural dorso‐ventral inversion in parapodial (and other) morphology in the sabellid thorax appears to be correlated with a physical separation between the ventral body wall and the nerve cord.

The correlation between the proximity of the nerve cord to the ventral body wall and polarity inversion is consistent with the hypothesis that the inversion in morphology of several structures in the sabellid thorax is related to, and possibly even caused by, a decreased influence of the nerve cord on the ventral body wall in the thorax, leading to a dorsalization of the ventral body wall in this region. This decreased proximity between nerve cord and body wall is similar to the effect of nerve cord ablation in nereids, which induces the dorsalization of the ventral body. Testing this hypothesis experimentally in sabellids, as was done in nereids, was not originally possible because the sabellid species used for these experiments (*Sabella pavonina* Savigny) were found not to survive well when removed from their tubes and surgically manipulated, precluding grafting experiments (B. Boilly, unpublished data). It is possible that other sabellid species may better tolerate the necessary experimental conditions, or that greater measures can be taken to prevent infection after surgery, allowing for testing of this hypothesis in this group of annelids. An alternative and potentially powerful approach for further investigating the origin of the DV polarity inversion in sabellids would be to identify and disrupt the molecular signals that establish DV polarity.

### 3.3 Epimorphic regeneration and morphallaxis

Sabellids have long been known to be able to regenerate anteriorly and posteriorly, with some species capable of doing so from nearly any segment (Berrill, 1978). In several sabellid species that have been studied, only a small number of segments are regenerated anteriorly, regardless of how many segments are removed (Berrill, 1931, 1978; Licciano, Murray, Watson, & Giangrande, 2012). In such species, a maximum of two to three segments are regenerated, by epimorphic regeneration (addition of new tissue), at the anterior wound site, and if more than that number are removed the epimorphic regeneration response is accompanied by a reorganization of the anterior‐most abdominal segments. This tissue reorganization, or morphallaxis, modifies the abdominal segments in situ such that they acquire the morphology of thoracic segments. When this process occurs in sabellids, morphallaxis involves a striking DV inversion of parapodia in the transformed segments and proceeds in an anterior to posterior direction until the initial number of thoracic segments is reconstituted (Berrill, 1978). Interestingly, a wave of morphallactic DV inversion has also been described in adult planaria when *bmp* signaling is disrupted; this process leads to the transformation of a normal animal into one with two ventral sides (Molina et al., 2011; Orii & Watanabe, 2007; Reddien et al., 2007).

Posteriorly, sabellid regeneration proceeds only by epimorphosis, in which new segments are added at the posterior wound site in an anterior to posterior progression. When regeneration begins from within the thorax, the stump regenerates the exact number of thoracic segments that are missing followed by a number of abdominal segments (Berrill, 1978). The posterior regenerative process can therefore reestablish the inversion in DV polarity along the AP axis that is characteristic in this group of annelids.

Given that the DV polarity of parapodia appears to be correlated with the proximity of the nerve cord to the body wall, it is tempting to propose that the nerve cord could be an important regulatory factor in DV patterning during morphallactic regeneration, and tissue reorganization more broadly, in sabellids. Consistent with this hypothesis, unpublished data by Sperry (1971), described in Berrill (1978), indicate that if the nerve cord is removed from 12 abdominal segments in *Bispira melanostigma* the denervated abdominal segments are transformed into thoracic‐like segments, acquiring an inverted DV polarity. These results in sabellids resemble those in nereids in which nerve cord deviation leads to a nerve‐cord‐less region which regenerates a completely dorsalized blastema (see above), although in Sperry's experiments segment regeneration was not involved since the worms were not transected. In both the sabellid and the nereid cases, then, the ventral side of denervated segments becomes dorsalized, lending further support to the hypothesis that the nerve cord is involved in establishing DV patterning in these annelids.

## 4 CONCLUSIONS AND FUTURE STUDIES

The studies reviewed here demonstrate that in two disparate groups of annelids, nereids and sabellids, the ventral nerve cord is implicated in post‐embryonic DV patterning. A series of experimental studies in nereids (involving a broad range of surgical manipulations and regeneration assays), a more limited number of experimental studies in sabellids, and correlational evidence in both groups all suggest that the nerve cord induces ventral fate, specifically of the body wall, in adult animals. Given that nereids and sabellids are distantly related annelids, representing the two major annelid clades (Errantia and Sedentaria, respectively) (Struck et al., 2011), the consistent findings from these two groups suggest that involvement of the ventral nerve cord in inducing ventral fate could be widespread and possibly ancestral for the phylum.

**Figure 12:**
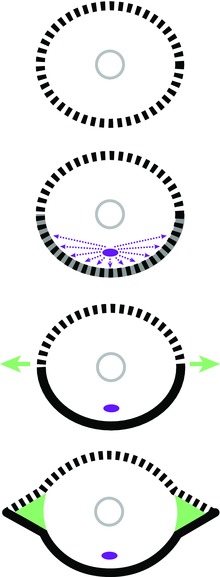
The nerve‐cord induction model for the establishment of dorso‐ventral polarity and parapodia in annelids. This model proposes that initially (top) the body wall has a default assignment of dorsal polarity (dashed) and that the nerve cord (purple) induces body wall in close proximity to take on a ventral polarity (darkened body wall). Contact between body wall of dorsal polarity and body wall of ventral polarity leads to body outgrowths (green arrows) at the position of DV discontinuity, from which develop the parapodia (green triangles)

More specifically, the experimental and correlational evidence from nereids and sabellids suggests the following: (i) the body wall has a default assignment of dorsal polarity, (ii) the body wall in proximity to the nerve cord becomes ventralized (leading to a polarity discontinuity between the dorsal and the ventral sides of the worm), and (iii) contact between body wall with ventral polarity and body wall with dorsal polarity causes the development of parapodia at the site of polarity discontinuity (Fig. 12). According to this model, then, several tissue interactions, specifically between the ventral nerve cord and the body wall and between ventral body wall and dorsal body wall, are key to the patterning and development of fundamental features of the annelid body.

Placing the annelid data reviewed here in the context of studies performed in other animals reveals several common elements. Most broadly, the studies reviewed here point to a central role for the nervous system in regeneration in annelids, as has been indicated for regeneration in a range of other animals (Kumar & Brockes, 2012; Pirotte et al., 2016). Another important common finding is that a DV discontinuity elicits body outgrowths. The annelid studies reviewed here indicate that, without the nerve cord, the body wall has a dorsal value in terms of positional information, as defined by Wolpert (1969). The dorsal value can be considered as the default quality because it is the only quality manifested in aneurogenic regenerates, while it is restricted to the dorsal area in innervated regenerates. The annelid data further suggest that the DV discontinuity between dorsal body wall and ventral body wall is what induces the lateral outgrowth of the parapodia. Outgrowths being induced at a site of DV discontinuity have also been demonstrated in other animals (reviewed by Boilly, Lheureux, Boilly‐Marer, & Bart, 1990). For example, DV discontinuity has been shown to play a fundamental role in initiating regeneration of the primary body axis (i.e., head/tail regeneration) in several planarians (Kato et al., 1999, 2001; Santos, 1931; Schilt, 1970) and in the control of morphogenesis during appendage regeneration in insects and limb regeneration in amphibians (reviewed by Bryant, French, & Bryant, 1981; Campbell & Crews, 2008; Endo, Bryant, & Gardiner, 2004). Studies are beginning to reveal the molecular basis of the role of innervation and of the positional information required for regeneration. For example, a recent study of limb regeneration in amphibians suggests that, after nerves at the wound site release trophic factors, regeneration‐competent cells at the wound site receive positional information mediated by the extracellular matrix, position‐specific heparan sulfate, and fibroblast growth factors (Phan et al., 2015).

An important next step for understanding the establishment of DV polarity in annelids is to identify the molecular signals underlying this process. Across a broad range of animals, DV polarity establishment involves some common molecular signals, most notably from homologs of BMP and BMP antagonists (de Robertis, 2008; de Robertis & Sasai, 1996). These molecular signals are thus prime candidates for being involved in the tissue patterning and interactions reviewed here in nereid and sabellid annelids. BMP signaling is implicated in DV specification not only during embryogenesis but during post‐embryonic development as well, such as in adult and regenerating platyhelminths and acoels. In planarians and acoel adults, orthologs of BMPs and *smad* (intracellular mediators of BMPs) are expressed on the dorsal side of the animal (Molina et al., 2007, 2011; Orii & Watanabe, 2007; Reddien et al., 2007; Srivastava et al., 2014) and orthologs of the BMP antagonists *admp* or *noggin* are expressed on the ventral side of the animal (Gavino & Riedden, 2011; Molina et al., 2007, 2011). Furthermore, a functional role of these genes in establishing DV polarity has been demonstrated by gene knockdown using RNA interference: knockdown of *bmp* orthologs in intact and regenerating planarians ventralizes the dorsal side, inducing an incomplete nerve cord on the dorsal side (Molina et al., 2011; Orii & Watanabe, 2007; Reddien et al., 2007). As has been found in several other studies, expression of *bmp* and *admp* orthologs appears to be spatially opposed in both planarians (Gavino & Reddien, 2011; Molina et al., 2011) and acoels (Srivastava et al., 2014). Interestingly, in planarians knockdown of a *noggin* ortholog induces dorsalization along the ventral midline (Molina et al., 2011), a result similar to that obtained in aneurogenic regenerates of nereids, reviewed above. Orthologs of *noggin* are thus potential candidates for being effectors of the ventralizing nerve cord signals in annelids. Although the effects of knockdowns of other ventral markers (e.g., Admp) are yet to be determined in regenerating planarians, BMP antagonists in planarians have been shown to be expressed in the nerve cord (Molina et al., 2007, 2011) or close to this structure (Gavino & Reddien, 2011). Given the involvement of BMP and BMP antagonists in DV establishment in a diverse array of animals, including during regeneration, future studies should test whether BMP signaling is involved in mediating the polarizing effects of the nerve cord in annelids.

Studies of BMP signaling are limited in annelids and have focused only on embryonic processes, yet available data suggest that these molecular signals are involved in DV establishment in this group of animals. In embryos of the clitellate annelid *Helobdella*, several homologs of BMP and a homolog of gremlin, a BMP antagonist, are expressed differentially along the DV axis (Kuo & Weisblat, 2011). Furthermore, functional studies indicate that BMP and gremlin homologs influence one another's expression and are indeed involved in DV patterning, with BMPs promoting dorsal fate and gremlin promoting ventral fate (Kuo & Weisblat, 2011). BMP signaling is thus implicated in DV patterning in *Helobdella*, although this appears not to hold true for the anterior‐most segments (Kuo, Shankland, & Weisblat, 2012). Orthologs of Admp, another BMP antagonist, have also been identified from *Helobdella* as well as from the polychaete annelid *Capitella teleta* (Gavino & Reddien, 2011; Kuo & Weisblat, 2011), although the mRNA does not appear to be differentially expressed along the DV axis in *Helobdella* (Kuo & Weisblat, 2011). BMP and BMP antagonists are thus present in annelids and some homologs appear to be functionally involved in establishing DV polarity during embryogenesis. BMP signaling has also been implicated in DV patterning in the embryos of molluscs (Lambert et al., 2016), which are relatively close relatives of annelids. Effectors of BMP signaling are thus obvious candidates for being involved in mediating the ventralizing effect of the nerve cord during annelid growth and regeneration. The hypothesis that a circuit involving BMPs and BMP antagonists is involved in DV establishment during post‐embryonic processes should be tested by investigating the expression of RNA and protein expression of these genes during regeneration and in surgically manipulated animals, as well as through functional studies that test the developmental consequences of altering the normal expression of these genes. If DV polarity can be inverted in regenerating adult animals by molecular disruptions of BMP signaling, this would constitute the clearest evidence that this signaling pathway regulates DV polarity during post‐embryonic development in annelids.

The establishment of DV polarity is a fundamental step in the development of bilaterian animals that can occur during embryogenesis as well as during post‐embryonic processes such as regeneration. Although some shared elements of DV polarity establishment are suggested by comparative studies, more work is needed to understand the conserved and divergent mechanisms by which dorsal and ventral fates are established in new tissues. Most studies focus on DV establishment during embryogenesis and more studies are needed of DV establishment during post‐embryonic processes such as adult growth and regeneration. Experimental studies in annelids reviewed here implicate the nerve cord in conferring ventral identity during adult development and regeneration and thereby suggest a number of hypotheses that should be tested further. Future studies that are likely to be particularly informative for understanding DV establishment in adult annelids include identifying the molecular players of DV establishment in growing and regenerating annelids (through both candidate gene and gene discovery approaches), determining the source of the key molecular signals (e.g., are molecular signals produced by the nerve cord itself or by neighboring cells?), determining the spatial range of such molecular signals, testing at the molecular level the hypothesis that body wall has a default dorsal identity, and identifying the molecular signals that induce outgrowth (e.g., parapodial outgrowth, blastema formation) at locations of DV discontinuity in annelids. With the increasing focus on annelid regeneration and with powerful new developmental and genetic tools becoming available for these animals (Ferrier, 2012; Özpolat & Bely, 2016; Seaver, 2016; Williams & Jékely, 2016; Zantke, Bannister, Veedin Rajan, Raible, & Tessmar‐Raible, 2014), such studies are now within reach and should provide important insights into the developmental mechanisms involved in establishing body polarity.
